# Impaired mitochondrial calcium uptake caused by tacrolimus underlies beta-cell failure

**DOI:** 10.1186/s12964-017-0203-0

**Published:** 2017-11-13

**Authors:** Angela Lombardi, Bruno Trimarco, Guido Iaccarino, Gaetano Santulli

**Affiliations:** 1 Department of Medicine, Albert Einstein College of Medicine, New York, NY USA; 2 Department of Advanced Biomedical Sciences, “Federico II” University of Naples, Naples, Italy; 3 Department of Medicine, Surgery and Dentistry, “Scuola Medica Salernitana”, University of Salerno, Fisciano, Italy

**Keywords:** Mitochondrial calcium, ATP, Diabetes, Insulin release, Immunosuppressive regimen, Ca^2+^ leak

## Abstract

**Background:**

One of the most common side effects of the immunosuppressive drug tacrolimus (FK506) is the increased risk of new-onset diabetes mellitus. However, the molecular mechanisms underlying this association have not been fully clarified.

**Methods:**

We studied the effects of the therapeutic dose of tacrolimus on mitochondrial fitness in beta-cells.

**Results:**

We demonstrate that tacrolimus impairs glucose-stimulated insulin secretion (GSIS) in beta-cells through a previously unidentified mechanism. Indeed, tacrolimus causes a decrease in mitochondrial Ca^2+^ uptake, accompanied by altered mitochondrial respiration and reduced ATP production, eventually leading to impaired GSIS.

**Conclusion:**

Our observations individuate a new fundamental mechanism responsible for the augmented incidence of diabetes following tacrolimus treatment. Indeed, this drug alters Ca^2+^ fluxes in mitochondria, thereby compromising metabolism-secretion coupling in beta-cells.

## Background

Tacrolimus (also known as fujimycin and FK506) is a macrolide lactone isolated from *Streptomyces tsukubaensis*, currently used as potent immunosuppressant in organ transplantation to reduce rejection rates [1, 2]. One of its most common adverse effects is new-onset diabetes mellitus following transplantation [3–7], a serious complication that also increases the risk of infection and cardiovascular disease [8]. Indeed, a 5-year follow-up study monitoring patients treated with tacrolimus after transplant revealed an incidence of diabetes of 41% [9].

The exact mechanisms underlying the diabetogenic effects of tacrolimus have not been fully elucidated. Various studies have suggested that tacrolimus side effects are attributable to its peripheral action, engendering a markedly reduced insulin sensitivity [10–12]. We hypothesize that tacrolimus at therapeutic dosage has also a direct detrimental effect on beta-cells, in particular on mitochondrial Ca^2+^ dynamics. To test our hypothesis, we evaluated the specific effects of tacrolimus on beta-cells.

## Methods

### Cell culture and drugs

INS-1 beta-cells (AddexBio, San Diego, CA) were cultured in a humidified atmosphere (37 °C) containing 5% CO_2_ in RPMI-1640 medium, and insulin levels were determined as previously described and validated by our group [13–15]. In some experiments, cells were treated with glucose (Bio-Techne, Abingdon, UK), L-leucine and glutamine (both from MyBioSource, San Diego, CA, USA), KCl (Merck KGaA, Darmstadt Germany) or tacrolimus (LC Laboratories, Woburn, MA, dissolved in dymethylsulfoxide).

### Cell viability assay

Cell viability was evaluated using the [3-(4,5-dimethylthiazol-2-yl)-2,5-diphenyl tetrazolium bromide (MTT) assay, as described [16, 17].

### Ca^2+^ measurements

Ca^2+^ imaging experiments were carried out as previously described [15, 18–20]. Briefly, to assess mitochondrial Ca^2+^, cells were loaded with Rhod-2 AM (3 μM, Thermo Fisher Scientific, Waltham, MA) at 37 °C for 30 min, followed by washout and 1 h rest at room temperature for de-esterification. Because of its delocalized positive charge, Rhod-2 AM accumulates preferentially within the mitochondrial matrix, where it is hydrolyzed and trapped [15, 20, 21]. Fluorescence was detected using a pass-band filter of 545–625 nm in response to excitation at 542 nm. Ca^2+^ mobilization from the ER following caffeine (10 mM, Biorbyt, Cambridge, UK) stimulation was assessed loading the cells with Fura-2 acetoxymethyl (AM) ester (Thermo Fisher Scientific, 5 μM, 15 min, 37 °C), as described [15, 18]; images were obtained using a dual excitation fluorescence imaging system: changes in intracellular Ca^2+^ were reflected in the ratio of fluorescence emission acquired above 510 nm in response to excitation at 340 nm and 380 nm. Intracellular Ca^2+^ leak was assessed spectrophotometrically in microsomes obtained from pancreatic beta-cells, as previously described [15]. Besides the above mentioned indirect evaluation of ER Ca^2+^ in response to caffeine, ER Ca^2+^ content was assessed using the FRET-based camaleon D1ER (Addgene, Cambridge, MA) [22].

### Mitochondrial respiration

Mitochondrial respiration was assessed using the Seahorse Analyzer (Agilent Technologies, Santa Clara, CA, USA), adding to each well glucose (16.7 mM), oligomycin (1 μM, Merck KGaA), carbonyl cyanide 4-(trifluoromethoxy) phenylhydrazone (FCCP, 0.5 μM, Merck KGaA), rotenone and antimycin A (both 1 μM, Merck KGaA). After each assay, cells were collected to quantify DNA using QuantiFluor dsDNA System (Promega, Madison, WI, USA).

### Cytochrome c oxidase activity assay

The cells were permeabilized by freeze-thaw cycle three times and mixed with detergent solution (0.1% bovine serum albumin, 250 mM sucrose, 10 mM KH_2_PO_4_ 2.5 mM laurylmaltoside, all from Merck KGaA). The enzymatic activity of cytochrome c oxidase was spectrophotometrically measured at 550 nm.

### ATP/ADP measurement

The genetically encoded sensors PercevalHR and pHRed (Addgene) were co-transfected in the cells in order to measure relative intracellular changes in ATP/ADP and pH, respectively. PercevalHR was excited at 405 nm (ADP) and 488 nm (ATP) and emission was collected at 530 nm. pHRed was excited using 405 nm and 546 nm and emission was collected at 630 nm band pass filter. Since PercevalHR has been reported to be sensitive to pH [23], a correction for pH was performed.

### Statistical analysis

Experiments were performed in a blinded fashion at least three times, unless otherwise noted. All results are presented as mean ± SEM. Statistical analysis was performed via unpaired *t* test when comparing two groups (normal distribution was confirmed by Anderson-Darling test) and one-way ANOVA, followed by Tukey-Kramer post hoc correction, when comparing more than two groups. A *P* value <0.05 was considered statistically significant.

## Results

### Glucose-induced insulin secretion (GSIS) is reduced following tacrolimus treatment

**Fig. 1:**
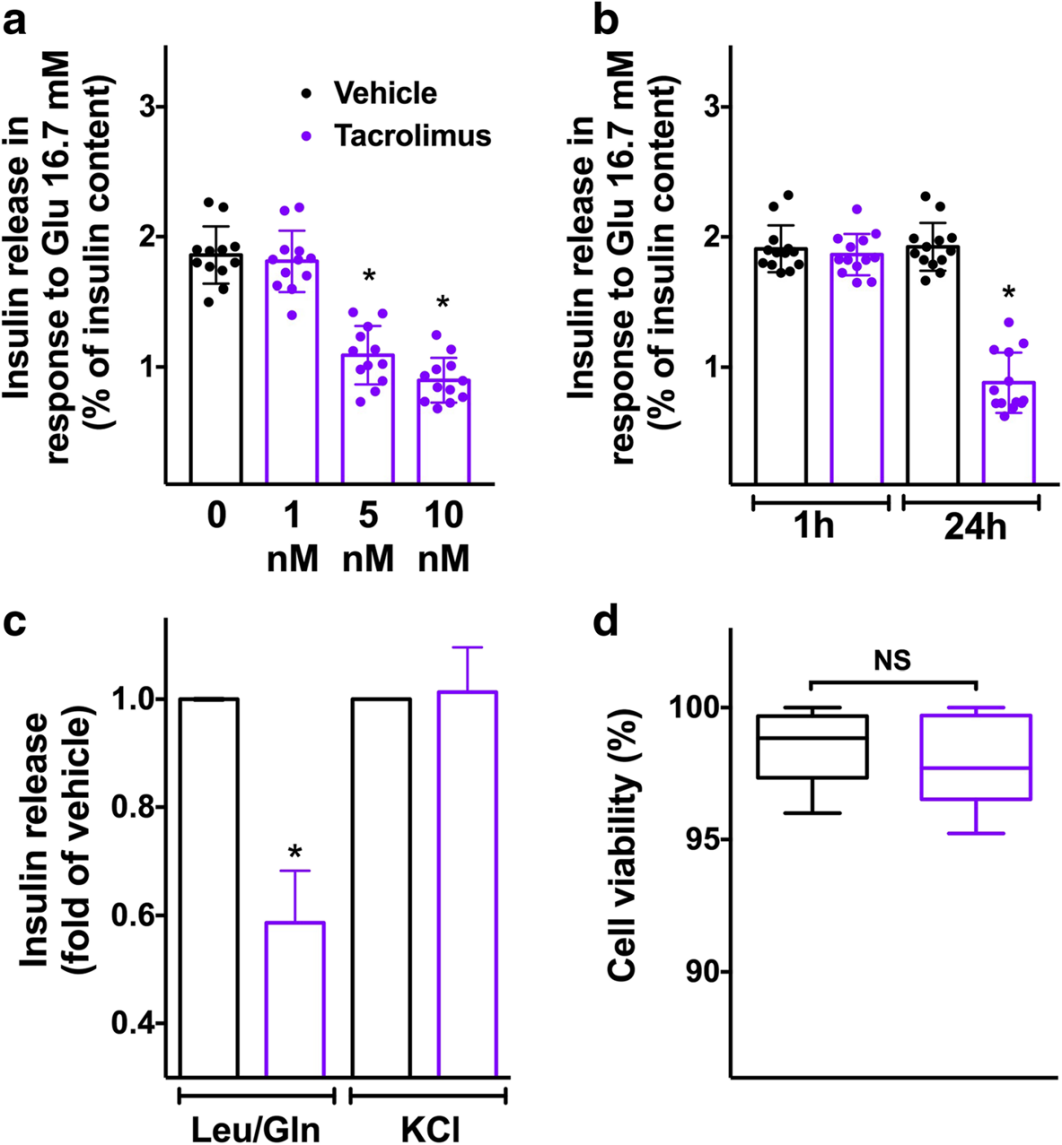
Therapeutic doses of tacrolimus alter insulin secretion without affecting cell viability. Insulin release measured in beta-cells incubated for 24 h with vehicle or tacrolimus at the indicated doses (**a**) and with vehicle or 5 nM tacrolimus at the indicated times (**b**). Effect of fuel secretagogues leucine (Leu, 10 mM) and glutamine (Gln, 2 mM) on beta-cells treated for 24 h with vehicle or 5 nM tacrolimus (**c**). Cell viability assessed in beta-cells treated for 24 h with vehicle or 5 nM tacrolimus (**d**). Data are presented as mean ± S.E.M. of experiments performed at least in triplicate; *:*p* < 0.01 vs vehicle, ANOVA. In panel **d**, whiskers represent 1% to 99% spread of the data; NS: non-significant; *t* test

To evaluate the effects of tacrolimus on beta-cell function, we measured GSIS in INS-1 beta-cells. In a dose-response assay, we found that the treatment with 5 nM tacrolimus, which is the average level that has been measured in the blood of patients undergoing organ transplantation [24], was sufficient to determine a significant reduction of GSIS compared with vehicle (Fig. 1a). The same dose (5 nM) was then tested in a time-course assay, which revealed that 24-h incubation markedly impaired GSIS (Fig. 1b). Importantly, tacrolimus had a significantly detrimental effect on the metabolic response of beta-cells to leucine and glutamine (Fig. 1c), strongly suggesting an alteration in mitochondrial oxidative metabolism. Supporting this view, insulin secretion in response to cell depolarization obtained via KCl, thereby bypassing mitochondria, was comparable between groups (Fig. 1c).

### Therapeutic doses of tacrolimus do not affect beta-cell viability

To test the effect of 5 nM tacrolimus on cell viability, we performed an MTT assay and we did not observe any significant effect on beta-cell viability (Fig. 1d).

### Tacrolimus alters mitochondrial Ca^2+^ uptake and mitochondrial respiration in beta-cells

**Fig. 2:**
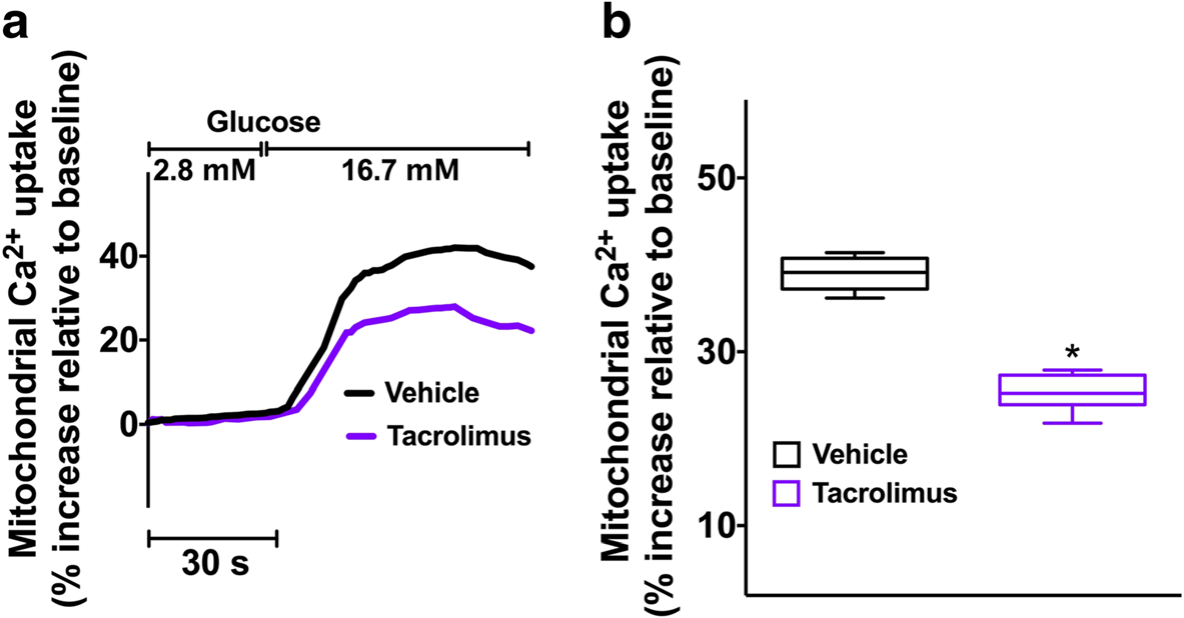
Tacrolimus impairs mitochondrial Ca^2+^ uptake. Representative traces (**a**) of glucose-induced mitochondrial Ca^2+^ uptake evaluated in beta-cells incubated for 24 h with vehicle or 5 nM Tacrolimus. **b** quantification of experiments (at least 35 cells per group were analyzed): whiskers represent 1% to 99% spread of the data; *:*p* < 0.001 vs vehicle

**Fig. 3:**
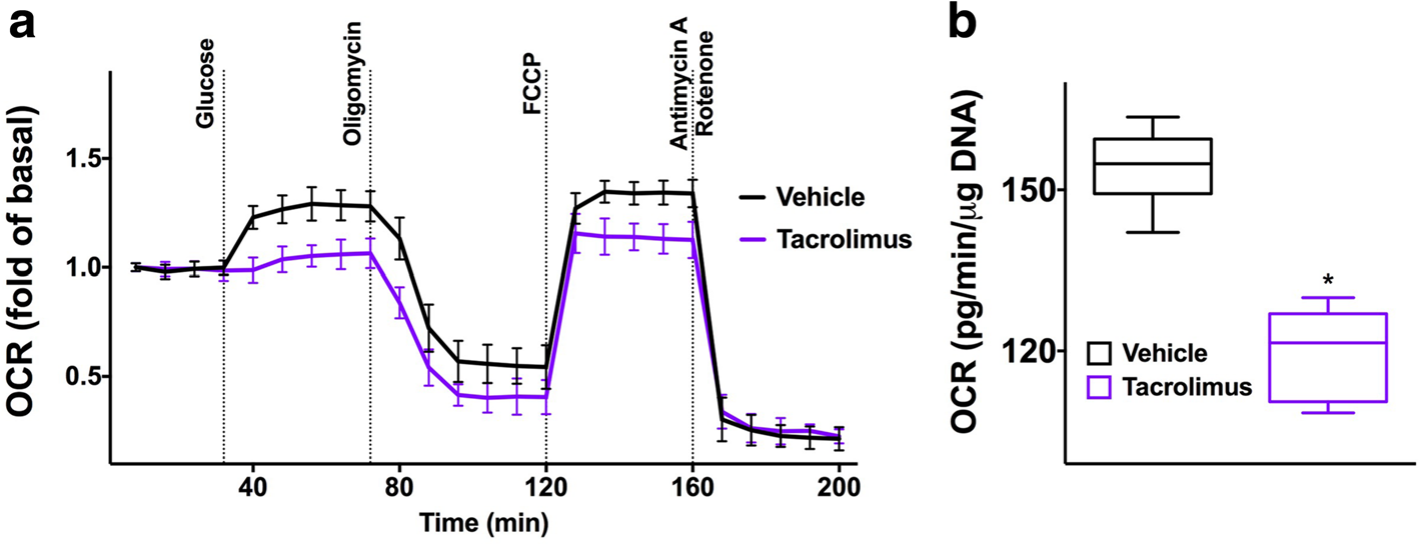
Tacrolimus impairs mitochondrial respiration in beta-cells. Analysis of oxygen consumption rate (OCR) in beta-cells incubated for 24 h with vehicle or 25 nM tacrolimus and then treated with glucose, oligomycin, phenylhydrazone (FCCP), antimycin A and rotenone, at the indicated times (**a**). Data represent mean ± S.E.M. of at least 4 independent experiments. **b** Quantification of the maximal respiratory capacity: whiskers indicate 1% to 99% spread of the data; *:*p* < 0.01 vs vehicle

Since mitochondrial Ca^2+^ has been shown to be a major determinant of beta-cell function, especially in terms of generation of metabolic coupling factors [25, 26], we evaluated the effect of tacrolimus on mitochondrial Ca^2+^. We found that tacrolimus significantly impairs mitochondrial Ca^2+^ uptake in beta-cells (Fig. 2a-b). Next, we assessed mitochondrial oxygen dynamics and we observed a marked decrease in oxygen consumption rate following incubation with tacrolimus (Fig. 3a-b), further supporting our hypothesis of a direct effect of this immunosuppressant on mitochondrial fitness.

### Tacrolimus decreases cytochrome c oxidase activity and causes a decrease in ATP/ADP ratio in beta-cells

**Fig. 4:**
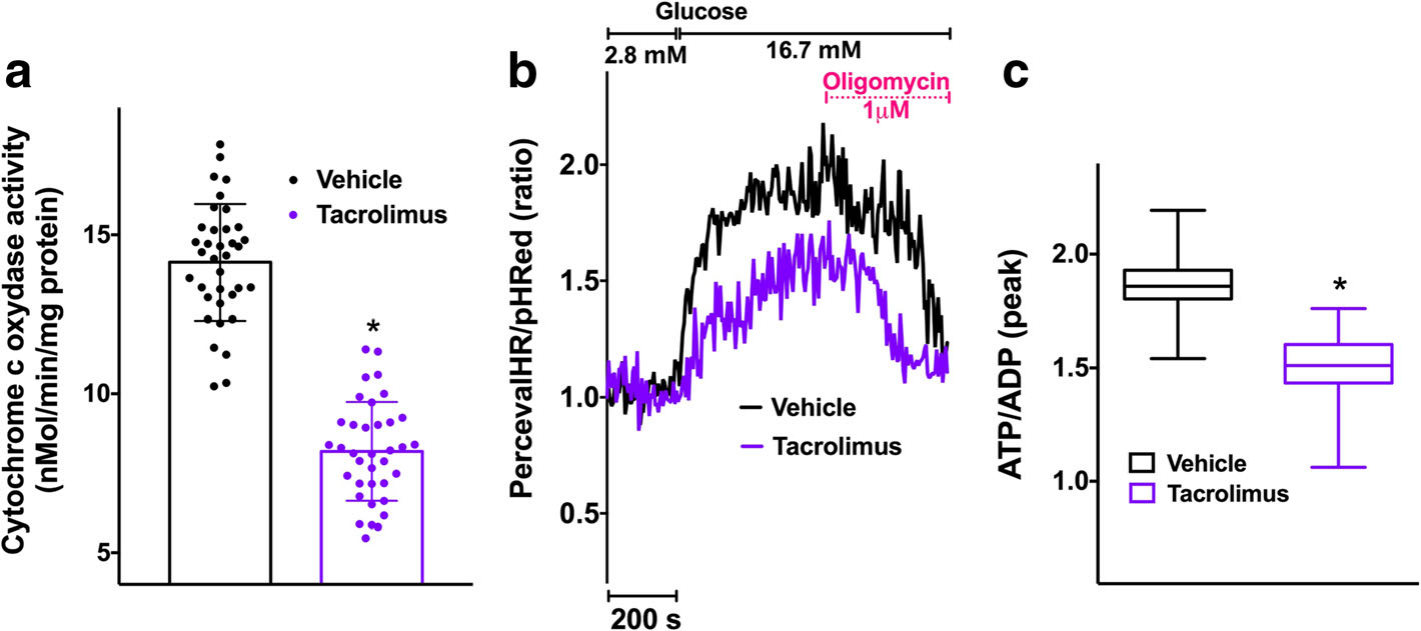
Tacrolimus at therapeutic dosage reduces mitochondrial ATP production. The enzymatic activity of cytochrome c oxidase was measured in beta-cells (at least 30 per group) incubated for 24 h with vehicle or 5 nM Tacrolimus (**a**); data represent mean ± S.E.M. The ATP/ADP ratio in response to 16.7 mM glucose was determined by PercevalHR ratio and corrected by pHRed ratio (**b**); the peak amplitude is quantified in panel **c**, where whiskers represent 1% to 99% spread of the data; *:*p* < 0.01 vs vehicle

To further investigate the role of tacrolimus on mitochondrial function, we first measured the enzymatic activity of cytochrome c oxidase, a key component of the electron transport chain; compared with vehicle-treated cells, we detected a significant reduction in cytochrome c oxidase activity following incubation with tacrolimus (Fig. 4a). Then, we evaluated the dynamics of ATP/ADP levels within the cell, fundamental regulators of fuel-stimulated insulin release [27]. Interestingly, tacrolimus significantly reduced ATP/ADP ratio in response to glucose compared with vehicle-treated cells (Fig. 4b-c).

### Tacrolimus at therapeutic doses depletes intracellular Ca^2+^ stores

**Fig. 5:**
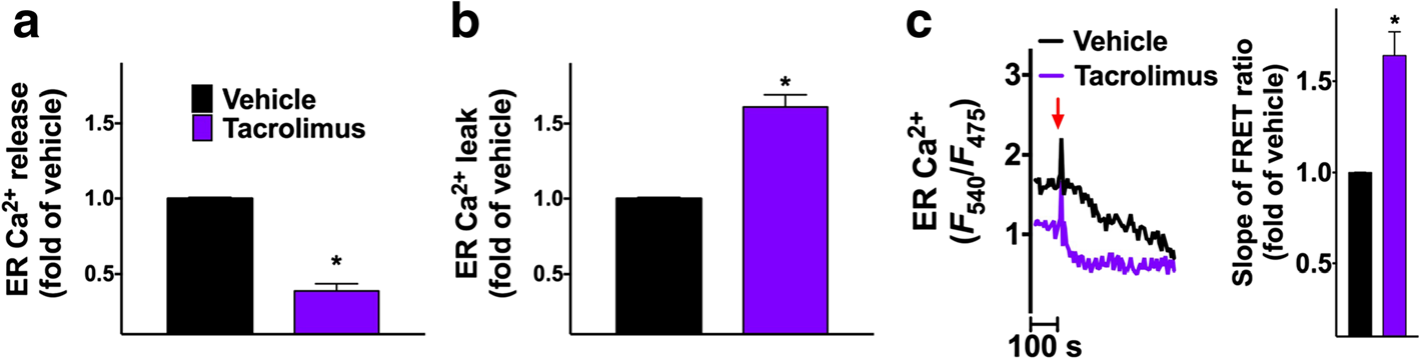
Tacrolimus causes ER Ca^2+^ depletion and ER Ca^2+^ leak. Endoplasmic reticulum (ER) Ca^2+^ stores (maximal peak amplitudes of caffeine-induced Ca^2+^ release, (**a**) and ER Ca^2+^ leak (**b**) were assessed following 24 h incubation with vehicle or 5 nM tacrolimus. **c** Direct evaluation of ER Ca^2+^ (representative curves, the arrow indicates thapsigargin, 1 μm) using the ER-targeted probe D1ER (left). The slope of the FRET ratio decrease after thapsigargin addition represents a bona fide measurement of the rate of Ca^2+^ leak (right), consistent with the results depicted in panel **b**. Data are presented as mean ± S.E.M. of at least 5 experiments performed in triplicate; *:*p* < 0.05 vs vehicle

Given the anatomical and functional connection between mitochondria and endoplasmic reticulum (ER) [28–31], we tested the effects of tacrolimus on ER Ca^2+^ dynamics, and we observed that 24 h incubation with 5 nM tacrolimus caused significant ER Ca^2+^ depletion and intracellular Ca^2+^ leak, evaluated both indirectly, assessing Ca^2+^ dynamics in response to caffeine, and directly, using an ER-targeted probe, with consistent results (Fig. 5).

## Discussion

The main finding of the present study is that tacrolimus has a detrimental effect on mitochondrial Ca^2+^ dynamics and beta-cell function, determining a reduced mitochondrial Ca^2+^ uptake and significantly impairing GSIS. Our results are consistent with the previous demonstration by Wollheim and colleagues of the essential role played by mitochondrial Ca^2+^ in metabolism-secretion coupling [26]. Indeed, one of the key roles of Ca^2+^ within mitochondria is to serve as a signal for oxidative metabolism, activating at least three biochemical reactions in the Krebs cycle, producing reducing equivalents [32], thereby accelerating mitochondrial respiration and ATP generation [27].

Several studies investigating the potential mechanisms underlying the pathogenesis of new-onset diabetes following tacrolimus-based treatments had focused on insulin-resistance [8, 10, 33–37]. Alterations in peripheral insulin-signaling have been also reported in response to other immunomodulators, including rapamycin, cyclosporine A, and steroids [38–42]. In fact, ultrastructural analysis has revealed that cyclosporine A causes mitochondrial dysmorphology in renal allograft biopsies [43]. Herein, we sought instead to test the effects of tacrolimus in vitro, using the clonal INS-1 beta-cells, which provide one of the best systems to directly study the pharmacologic effects of a compound on beta-cell behavior, avoiding extracellular confounding factors and potential compensatory mechanisms present in whole organisms or in islet clusters [44], especially for the assessment of mitochondrial Ca^2+^ dynamics [26, 45–47]. Of course, we acknowledge that the lack of experimental evidence in islets or animal models can also be seen as a limitation of this work.

We demonstrate here that 5 nM tacrolimus is sufficient to determine a marked reduction in GSIS, without affecting cell viability. We decided to test such dose because this is the average level of drug that has been actually measured in islet transplant recipients [24]. The lack of significant effect of therapeutic doses of tacrolimus on cell viability is consistent with earlier observations in HIT-T15 hamster beta-cells [48]. Instead, when used at high doses (e.g. 1 μM) tacrolimus does cause apoptosis, as shown ex vivo in experiments performed on rat islets [49]. The pro-apoptotic action of tacrolimus following prolonged exposures (> 24 h) has been also reported [50].

We and others have previously shown that mitochondrial function in pancreatic beta-cells and other tissues is mechanistically related to intracellular Ca^2+^ fluxes [15, 28, 31, 32, 51]. Here we demonstrate that tacrolimus treatment directly impairs mitochondrial Ca^2+^ uptake. This phenomenon could explain the reduced mitochondrial respiration observed in tacrolimus-treated beta-cells. Indeed, as mentioned above, Ca^2+^ activates dehydrogenases in the tricarboxylic acid cycle within the mitochondrion [32].

We also demonstrate here that an impaired mitochondrial Ca^2+^ uptake is mechanistically linked to mitochondrial dysfunction, as shown by the reduced enzymatic activity of cytochrome c oxidase following tacrolimus treatment. Our findings are consistent with the decreased mitochondrial bioenergetics observed in rat islets incubated with tacrolimus [52]. Moreover, tacrolimus has been shown to potentiate glucolipotoxicity in different models [53]. Crucially, the dynamic evaluation of ATP/ADP levels confirms that tacrolimus-treated cells generate less ATP compared with vehicle-treated cells, an observation that is in line with earlier reports suggesting that mitochondrial Ca^2+^ is essential for energy production [54–56]. The decreased insulin release detected in tacrolimus-treated beta-cells following stimulation with secretagogues that trigger insulin secretion via mitochondrial metabolism (e.g. leucine/glutamine), but not following KCl-induced membrane depolarization, corroborates this view, strongly indicating a defect in mitochondrial bioenergetics. Further studies are necessary to decipher in detail the exact processes linking tacrolimus pharmacodynamics to the observed effects on ER and mitochondrial Ca^2+^ reported here for the first time. Potential candidates, currently being investigated in our laboratory, include the immunophilin FK506 binding protein (FKBP), which forms a complex with tacrolimus [57–59], and ER stress, which has been recently reported in tacrolimus-treated lymphocytes [60].

## Conclusion

Our findings individuate in impaired mitochondrial Ca^2+^ uptake a novel mechanism underlying the increased incidence of diabetes following tacrolimus treatment.

## Abbreviations

Ca^2+^: Calcium
ER: Endoplasmic reticulum
FCCP: Carbonyl cyanide 4-(trifluoromethoxy) phenylhydrazone
Fura-2 AM: Fura-2-acetoxymethyl ester
GSIS: Glucose-stimulated insulin secretion
Rhod2-AM: 1-[2-Amino-5-(3-dimethylamino-6- dimethylammonio- 9-xanthenyl) phenoxy]-2-(2-amino-5-methylphenoxy) ethane-N,N,N′,N′-tetra-acetic acid, tetra-acetoxymethyl ester
